# Spontaneous Subdural Hematoma in a Healthy 28-Year-Old Male During Latissimus Dorsi Pulldowns: A Case Report

**DOI:** 10.7759/cureus.85038

**Published:** 2025-05-29

**Authors:** Daniel I Razick, Ashley Yuen, Bernardo Gavidia, Cody Kaiser, Danielle Berera

**Affiliations:** 1 Surgery, California Northstate University College of Medicine, Elk Grove, USA; 2 Surgery, San Joaquin General Hospital, French Camp, USA

**Keywords:** craniotomy, latissimus dorsi pulldown, midline shift, non-traumatic subdural hematoma, subdural hematoma

## Abstract

Non-traumatic subdural hematomas (SDHs) in healthy, young individuals are rare, with strenuous physical activity occasionally implicated. We present such a case in a 28-year-old male with no pertinent past medical, surgical, or family history. The patient presented to the emergency department six days after he was performing latissimus dorsi pulldowns during a workout at approximately 3:00 AM when he experienced a sudden “pop” sensation in his head followed by transient loss of vision in his left eye for 20 minutes, dizziness, and nausea. The symptoms temporarily improved enough for him to drive home. However, he developed a persistent headache, nausea, and intermittent vomiting over the subsequent days. On day 6, intractable vomiting and persistent dizziness prompted emergency department evaluation. Initial non-contrast computed tomography (CT) of the head revealed an 8-mm left frontoparietal SDH, while CT angiography was unremarkable. He reported dehydration and consumption of a high-caffeine energy drink before his workout. He denied recreational or performance-enhancing drug use or trauma. He underwent left-sided craniotomy with SDH evacuation and subdural drain placement. Postoperative imaging confirmed hematoma evacuation and improved midline shift. Apart from transient confusion on postoperative day 3, his recovery was uneventful and he was discharged on postoperative day 5. He reported complete resolution of symptoms at a two-week follow-up. We review the available literature regarding non-traumatic SDHs and speculate the cause of SDH in our case.

## Introduction

A subdural hematoma (SDH) is a collection of blood between the dura and arachnoid mater, classically resulting from stretching or rupture of bridging veins. SDH most commonly occurs following blunt head injury, but it may also result from penetrating head injuries or non-traumatically via spontaneous vessel rupture [1]. Obtaining a thorough patient history and conducting a comprehensive physical examination are critical in the diagnosis of SDH. Patients may report a history of recent trauma, while symptoms vary depending on whether the SDH is acute, chronic, or acute chronic. Acute symptoms may include headache, nausea, vomiting, altered mental status, or seizure. Chronic SDH may present with progressive confusion, decreased consciousness, motor deficits, aphasia, or personality changes. Computed tomography (CT) without contrast is the preferred imaging modality in the diagnosis of SDH and characteristically demonstrates a crescent-shaped region of blood [2].

Management is dictated based on the size of the hematoma, the presence of midline shift, and neurological status. Treatment options include supportive care with close observation or surgical evacuation via burr hole drainage or craniotomy. If left untreated, the increase in intracranial pressure and potential midline shifting of the brain can progress to severe neurological deterioration [3]. Cases of non-traumatic SDH have been reported in association with intense physical exertion, coagulopathies, or spontaneous vascular rupture [4-6]. However, non-traumatic SDH is rare in young, healthy individuals. Given the unique nature of the case and limited literature regarding non-traumatic SDH, we present a case of non-traumatic SDH in a healthy 28-year-old male while performing latissimus dorsi pulldowns (lat pulldowns).

## Case presentation

A 28-year-old male with no pertinent past medical or family history presented to the emergency department six days after he was performing lat pulldowns during a workout at approximately 3:00 AM when he experienced a sudden “pop” sensation in his head followed by transient blindness in his left eye for 20 minutes, dizziness, and nausea. He states that at that time he concluded his workout and was able to drive himself home and slept for four hours. Over the next six days, he experienced intermittent headache, nausea, and vomiting until day 6 when he felt dizzy the entire day and experienced intractable vomiting, prompting his presentation to the emergency department.

**Figure 1:**
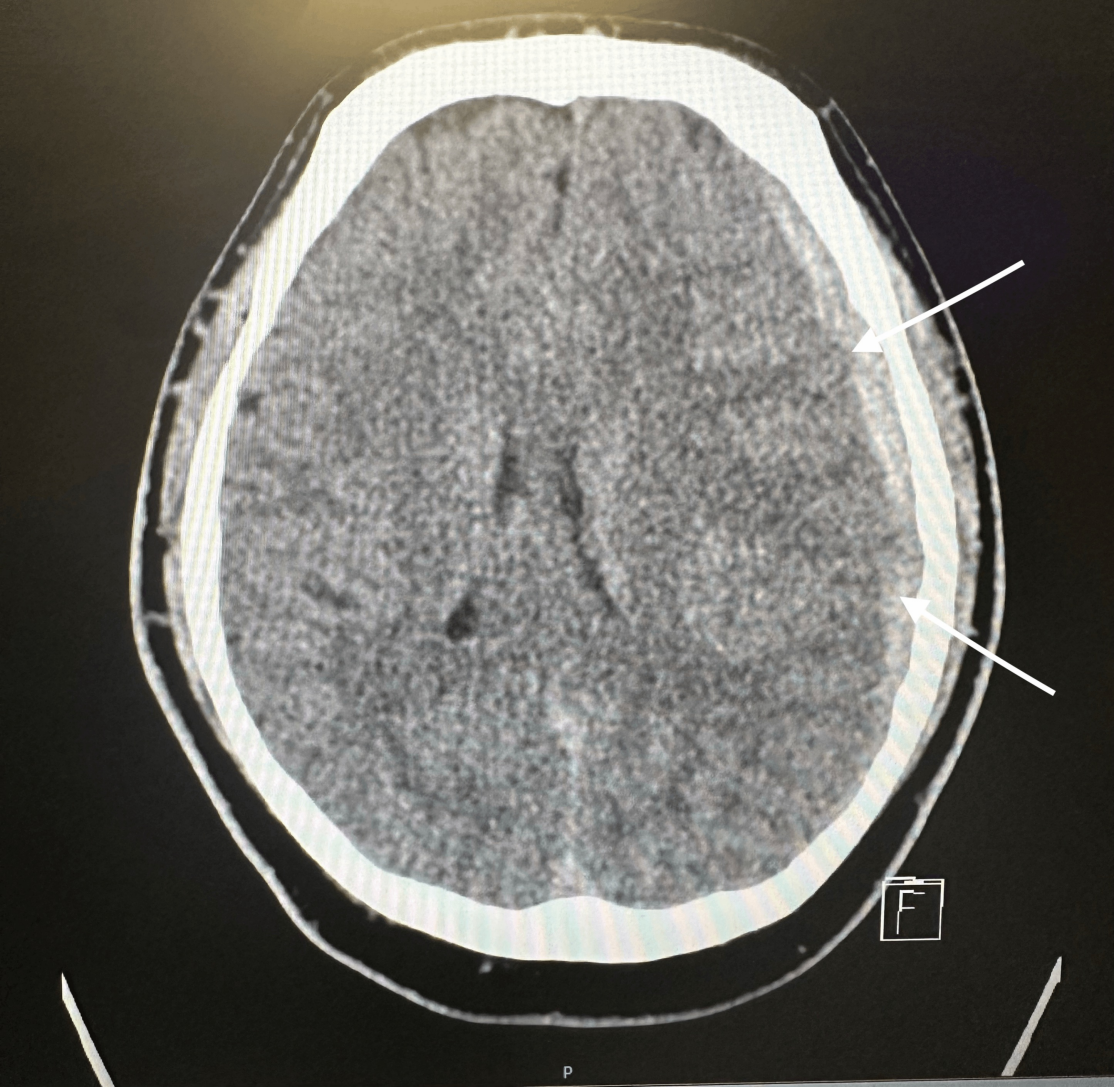
Transverse CT image prior to craniotomy demonstrating SDH CT, computed tomography; SDH, subdural hematoma

Upon arrival, the patient was awake and oriented, and the Glasgow Coma Scale (GCS) score was 15. There were no focal neurological deficits, though the patient was moderately unstable when ambulating. Blood pressure was elevated at 148/92, heart rate was 86 beats per minute, and respiratory rate was 12 breaths per minute. CT scan without contrast demonstrated an 8-mm left-sided, subacute frontoparietal SDH with a right-sided midline shift of 5.6 mm (Figure 1).

The patient denied numbness, tingling, or weakness in the upper or lower extremities. When asked about possible contributing factors, the patient stated that he felt dehydrated the day before the event and consumed a high-caffeine energy drink before his workout. The patient had been consistently engaging in weightlifting for one year and had lost 70 pounds over the year. His diet and workout regimen were unchanged recently. The patient stated that six months prior, he completely quit smoking marijuana. He reported smoking nearly 1 gm of marijuana daily for two years before quitting. He denied direct trauma to the head and stated that he was on his third set of lat pulldowns lifting 180 pounds, which he performed after completing four prior exercises. He denied taking anabolic steroids or other weight loss or muscle-building supplements. He denied taking additional medications.

**Figure 2:**
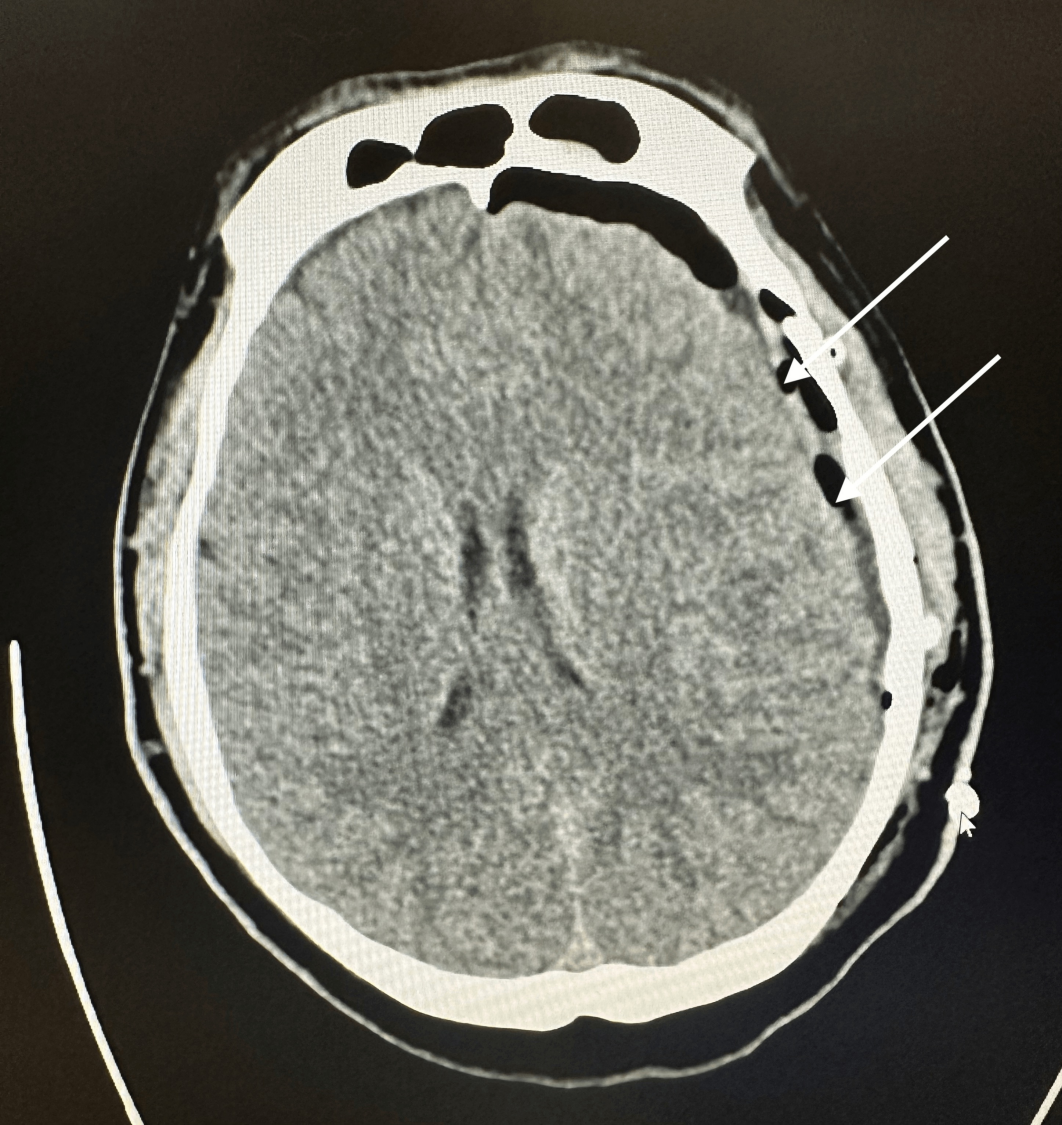
Transverse CT image post-craniotomy demonstrating resolution of SDH CT, computed tomography; SDH, subdural hematoma

Neurosurgery was consulted, which recommended craniotomy for evacuation of the hematoma; however, the patient did not elect to undergo surgery at that time. He was hesitant to undergo a major operation and opted for conservative management first. He was subsequently admitted to the hospital for close monitoring with neurological checks every 24 hours. A repeat CT scan was performed the next day, which showed a stable SDH with no new areas of hemorrhage. The patient was subsequently discharged and instructed to follow up in the clinic two days later. Upon follow-up in the neurosurgery clinic, the patient complained of significantly worsening headache, nausea, and vomiting. Given his worsening symptoms, the patient consented to surgery and underwent craniotomy with evacuation of the SDH and placement of a subdural drain later that day. Immediate postoperative CT scan showed resolution of the hematoma with reduced rightward midline shift (Figure 2). The patient reported a mild headache consistent with postoperative course but reported significant improvement in symptoms compared to his preoperative status. GCS score was 15 postoperatively.

The patient remained admitted for five days. He experienced one acute episode of confusion on postoperative day 3, which resolved without neurosurgical intervention and with no change in head CT scan findings. He was discharged on postoperative day 5 without neurological deficits. At the two-week follow-up, the patient reported a complete resolution of symptoms.

## Discussion

Non-traumatic SDH is rare, accounting for 0.7-6.7% of all acute SDHs [7]. Coombs et al. conducted a review of 193 cases of non-traumatic SDH and found the most common etiologies to be cortical artery bleeding, vascular lesions, neoplasms, and coagulopathy [8]. While the exact etiology of the SDH presented in the present case remains unclear, several factors may have contributed to its development.

Intense physical exertion has been implicated in cases of spontaneous SDH, likely due to acute increases in intracranial and intravascular pressure. A report by Davis et al. described the case of a 42-year-old woman who developed bilateral SDHs following a Pilates class [9]. Another study by Alaraj et al. described two cases of spontaneous SDH in anabolic steroid-dependent weightlifters but concluded that chronic anabolic steroid use likely induced vascular changes that predisposed the patients to bleed during Valsalva maneuver-like exercises [10]. Valsalva maneuvers have been shown to invoke transient increases in intracranial pressure and systemic blood pressure, with quick returns to baseline after cessation of the maneuver [11]. Interestingly, the patient described in the present case was performing lat pulldowns, a movement that has not been shown to increase intra-abdominal pressure or imitate Valsalva, nor was he taking anabolic steroids. It remains uncertain whether the patient was holding his breath while performing the exercise, which may have caused a rapid increase in intracranial pressure.

Another potential contributing factor in the pathogenesis of this patient’s SDH is his extended history of cannabis use. Though he reportedly quit smoking marijuana six months before the incident, long-term cannabis use has been associated with increased arterial stiffness and increased baseline heart rates, potentially predisposing patients to cerebrovascular events [12]. Although a direct association between cannabis use and SDH has not been established, the patient’s chronic marijuana consumption may have contributed to the development of vascular vulnerability.

The patient also reported feeling dehydrated before the event and consumed a high-caffeine energy drink several minutes before his workout began. It is well-known that hypohydration can increase blood pressure by activating the renin-angiotensin-aldosterone system (RAAS), while acute hypohydration impairs vascular function and blood pressure regulation [13]. Caffeine is a widely consumed neurostimulant and adenosine antagonist that induces cerebral vasoconstriction [14]. The combination of hypohydration, high levels of caffeine, and strenuous exercise may have created an environment conducive to venous rupture. Finally, while CT angiography did not reveal any vascular abnormalities, subtle vascular anomalies may have contributed to the development of SDH in this patient. Magnetic resonance imaging (MRI) or magnetic resonance angiography (MRA) should be considered to visualize vascular abnormalities if SDH recurs. Regardless of the etiology of this patient’s SDH, this case highlights the need for further investigation into possible predisposing factors in otherwise healthy, young individuals.

## Conclusions

This case highlights the importance of taking a comprehensive history and timely intervention to achieve favorable outcomes in SDH patients. In conclusion, non-traumatic SDH should be considered in young patients presenting with acute neurological symptoms following intense exercise, particularly weightlifting movements, which may increase venous pressure to the point of vein rupture. Surgical intervention can result in good outcomes despite delayed presentation. Further investigation into possible predisposing factors in otherwise healthy, young individuals is warranted.
